# Reduced Right Ventricular Native Myocardial T_1_ in Anderson-Fabry Disease: Comparison to Pulmonary Hypertension and Healthy Controls

**DOI:** 10.1371/journal.pone.0157565

**Published:** 2016-06-15

**Authors:** Joseph J. Pagano, Kelvin Chow, Aneal Khan, Evangelos Michelakis, Ian Paterson, Gavin Y. Oudit, Richard B. Thompson

**Affiliations:** 1 Department of Biomedical Engineering, University of Alberta, Edmonton, Alberta, Canada; 2 Department of Medical Genetics, University of Calgary, Calgary, Alberta, Canada; 3 Department of Medicine, Mazankowski Alberta Heart Institute, University of Alberta, Edmonton, Alberta, Canada; Cincinnati Children's Hospital Medical Center, UNITED STATES

## Abstract

**Aims:**

Anderson-Fabry disease (AFD) is characterized by progressive multiorgan accumulation of intracellular sphingolipids due to α-galactosidase A enzyme deficiency, resulting in progressive ventricular hypertrophy, heart failure, arrhythmias, and death. Decreased native (non-contrast) left ventricular (LV) T_1_ (longitudinal relaxation time) with MRI discriminates AFD from healthy controls or other presentations of concentric hypertrophy, but the right ventricle (RV) has not been studied. The aims of the current study were to evaluate native RV T_1_ values in AFD, with a goal of better understanding the pathophysiology of RV involvement.

**Methods and Results:**

Native T_1_ values were measured in the inferior RV wall (RVI), interventricular septum (IVS), and inferior LV (LVI) in patients with AFD, patients with pulmonary hypertension, who provided an alternative RV pathological process for comparison, and healthy controls. A minimum wall thickness of 4 mm was selected to minimize partial volume errors in tissue T_1_ analysis. T_1_ analysis was performed in 6 subjects with AFD, 6 subjects with PH, and 21 controls. Native T_1_ values were shorter (adjusted p<0.05 for all comparisons), independent of location, in subjects with AFD (RVI-T_1_ = 1096±49 ms, IVS-T_1_ = 1053±41 ms, LVI-T_1_ = 1072±44 ms) compared to both PH (RVI-T_1_ = 1239±41 ms, IVS-T_1_ = 1280±123 ms, LVI-T_1_ = 1274±57 ms) and HC (IVS-T_1_ = 1180±60 ms, LVI-T_1_ = 1183±45 ms). RVI measurements were not possible in controls due to insufficient wall thickness.

**Conclusion:**

**N**ative T_1_ values appear similarly reduced in the left and right ventricles of individuals with AFD and RV wall thickening, suggesting a common pathology. In contrast, individuals with PH and thickened RVs showed increased native T_1_ values in both ventricles, suggestive of fibrosis.

## Introduction

Anderson-Fabry disease (AFD), an X-linked lysosomal storage disease, is characterized by progressive multiorgan accumulation of intracellular sphingolipids due to α-galactosidase A enzyme deficiency[1, 2]. Cardiac involvement can result in progressive ventricular hypertrophy leading to heart failure, arrhythmias and is now the most common cause of mortality in patients with AFD[1, 2]. Due to inherent risks and limitations of endomyocardial biopsy[3], non-invasive measures are sought as surrogates for sphingolipid deposition[4, 5]. While the focus has been primarily on global changes in cardiac structure and function, such as increasing ventricular mass[6], atrioventricular uncoupling[7], and reduced myocardial function[8–10], recent studies using cardiac magnetic resonance T_1_-mapping techniques show promise in providing improved diagnostic differentiation between other causes of ventricular hypertrophy, as well as the prospect of an earlier marker of disease involvement[11–13].

Measurement of native myocardial T_1_ (longitudinal relaxation) time using cardiac magnetic resonance imaging (CMR) has revealed increased values in individuals with numerous cardiac conditions, including cardiomyopathies, acute myocarditis, and acute myocardial infarction[14–16]. In contrast, significantly reduced left ventricular (LV) T_1_ values have been measured in patients with AFD, with average values >100 ms lower than healthy subjects, and larger magnitude changes compared to other conditions presenting with similar LV hypertrophy[11, 12]. T_1_-mapping is thus a promising tool in differentiating distinct manifestations of hypertrophy and has been proposed as a quantitative biomarker to follow for response to therapy, such as enzyme replacement in AFD[11, 12].

While left ventricular hypertrophy is a hallmark of AFD, right ventricular (RV) involvement is also commonly seen, including ventricular hypertrophy and dysfunction[17–20]. Right ventricular dysfunction likely contributes to the presence of heart failure symptoms in those with preserved LV ejection fraction[17]. While autopsy studies have shown sphingolipid deposition in both ventricles[21], it is important to note that despite studies which have shown beneficial effects from enzyme replacement therapy on LV metrics, there have been inconsistent changes seen in the RV[19, 20]. This may indicate subtle differences in the pathophysiological mechanisms behind ventricular remodelling and dysfunction in Fabry disease. Due to the ability of native T_1_ to differentiate those with Fabry disease from other cases of LV hypertrophy, it offers a non-invasive metric that may help understand if the RV involvement mirrors that of the LV. However, the assessment of RV T_1_ values in AFD have not previously been reported. Thus, the goal of the current study was to evaluate quantitative T_1_-mapping in the RV of patients with AFD. To aid in the understanding of the underlying mechanism of RV involvement in AFD, RV T_1_ values were compared to LV values in patients with AFD and RV T_1_ values in a group of patients with idiopathic pulmonary hypertension (PH), in whom changes in T_1_ values are representative of increased RV afterload[22].

## Methods

### Subjects

The primary study patient cohort consisted of 32 subjects with clinically and genetically confirmed AFD and 11 subjects with pulmonary hypertension, in whom T_1_-mapping was performed as part of existing studies. Average left ventricular T_1_ values from the healthy controls and subjects with AFD have previously been published[12]. Subjects with AFD were recruited from both the University of Alberta and University of Calgary, from May 2010 to November 2012. Subjects were included if they had clinically and genetically confirmed AFD, and excluded if they were unable to provide informed consent or had contraindication to CMR. Subjects with PH were recruited from the University of Alberta from March 2010 to June 2013. Subjects were included if they had PH due to familial conditions, associated with anorexic medications, or idiopathic PH, and excluded if they were unable to provide informed consent, had abnormal renal function (GFR <30 mL/min/1.73 m^2^), or had a contraindication to CMR. Pulmonary hypertension was defined by catheter measured mean pulmonary artery pressure ≥ 25 mmHg, pulmonary capillary wedge pressure ≤ 15 mmHg, and pulmonary vascular resistance > 240 dynes·sec/cm^5^. Subjects with PH also needed to have had no changes within 2 months to medications approved for treatment of pulmonary hypertension, with stable New York Heart Association class of III-IV. Normal LV T_1_ values were obtained from a healthy control (HC) group from an ongoing study of heart failure with preserved ejection fraction (Alberta Heart Failure Etiology and Analysis Research Team [HEART])[23], who were recruited from the region around Edmonton, Alberta, between January 2010 and October 2014. Healthy controls had no evidence of heart disease or significant cardiovascular risk factors, including coronary artery disease, hypertension, diabetes mellitus, inflammatory or autoimmune diseases, and could not be on any cardiac medication or have contraindication to CMR. The studies were approved by the University of Alberta and University of Calgary health research ethics boards. Informed written consent was obtained from all subjects.

### CMR Imaging

CMR was performed on 1.5T systems (Siemens Sonata or Avanto, Siemens Medical Solutions, Erlangen, Germany). Short- and long-axis ventricular cines were performed using balanced steady-state free procession (bSSFP) imaging. Typical scan parameters were 1.24 ms echo time, 2.48 ms repetition time, 51° flip angle, 8 mm slice thickness, 2 mm gap, 400×275 mm field of view, 256×132 acquisition matrix, 75% phase resolution, 14 views per segment, rate 2 parallel imaging (GRAPPA), and 30 reconstructed cardiac phases. T_1_-mapping was performed on a mid-ventricular short-axis slice during diastasis using the SAturation-recovery single-SHot Acquisition (SASHA) pulse sequence with a bSSFP readout, as previously described[12, 24]. Typical SASHA parameters were 1.36 ms echo time, 2.72 ms repetition time, 70° flip angle, 9 images spanning 100–650 ms saturation recovery times plus a non-saturated image, 8 mm slice thickness, 360×270 mm field of view, 192×108 acquisition matrix before interpolation, and 75% phase resolution. Acquired in-plane spatial resolution was 1.9 mm, and was interpolated to 0.94 mm for analysis. Either rate 2 parallel imaging (GRAPPA) or 6/8 partial Fourier was used for image acceleration. A phase sensitive inversion recovery sequence was used for conventional late gadolinium enhancement (LGE) imaging, typically starting 7 minutes following gadolinium based contrast injection, with coverage matching cine locations. Typical LGE parameters were 4.18 ms echo time, 25° flip angle, 8 mm slice thickness, 380×285 mm field of view, 256×173 acquisition matrix, 90% phase resolution, and 25 views per segment. Subjects with AFD, along with healthy controls, received 0.15 mmol/kg gadobutrol (Gadovist; Bayer HealthCare Pharmaceuticals, Montville, NJ), while subjects with PH received 0.1 mmol/kg gadolinium-diethylenetriaminepentaacetic acid (Gd-DTPA, Magnevist; Bayer Healthcare, Toronto, Canada).

### Data Analysis

Manual endocardial tracing of both ventricles was performed for quantification of ventricular end-systolic volume and end-diastolic volume. Ejection fraction was calculated as (end-diastolic volume—end-systolic volume)/ end-diastolic volume. Manual epicardial tracing of the left ventricle, excluding papillary muscles, was performed for quantification of ventricular mass, calculated as the ventricular muscle volume corrected for specific gravity of the tissue (1.05 g/mL). Masses and volumes were indexed to body surface area.

Late gadolinium enhancement images were assessed for the presence of positive enhancement, with specific assessment of the interventricular septum, inferior RV wall, inferior LV wall, and the inferior RV insertion point at the slice location nearest to the corresponding T_1_-mapping images. Additional LGE short and long axis slices were used to corroborate the presence or absence of positive enhancement in these locations of interest. Nine subjects with Anderson-Fabry disease did not receive gadolinium contrast, either due to research protocol (n = 8) or renal dysfunction (n = 1).

**Fig 1:**
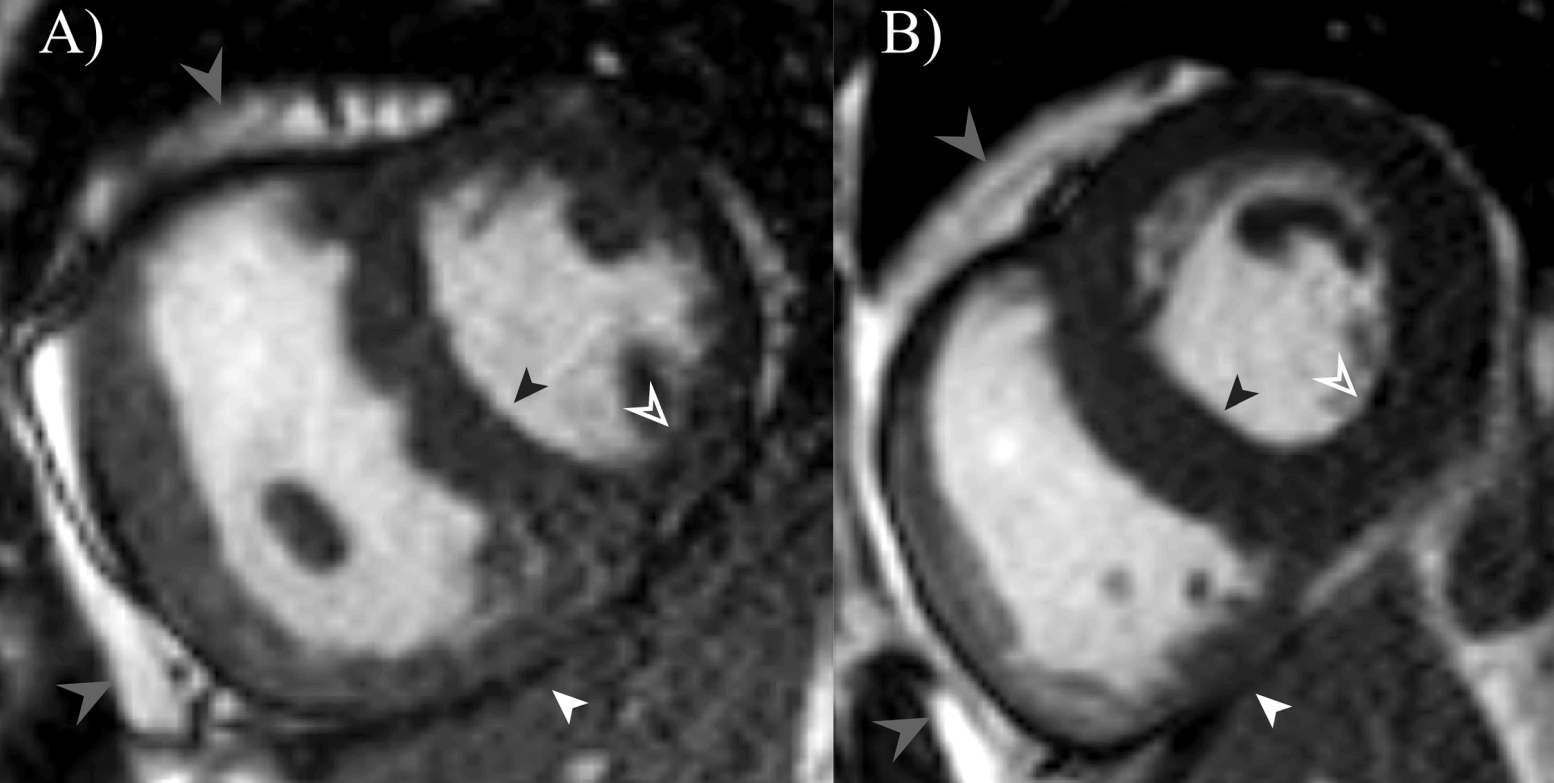
Example of bSSFP cine images in diastole used for wall thickness measurements. Measurement locations are shown at the inferior RV wall (RVI thickness, white arrowhead), interventricular septum (IVS thickness, black arrowhead), and inferior LV wall (LVI thickness, open arrowhead) for a subject with pulmonary hypertension (A) and Anderson-Fabry disease (B). Light grey arrows show areas of RV epicardial fat. Note the absence of readily visible epicardial fat along the inferior RV wall.

Inferior RV wall thickness (RVI thickness), interventricular septal wall thickness (IVS thickness), and inferior LV wall thickness (LVI thickness) was measured in all subjects at a diastasis cardiac phase on short-axis cine images (Fig 1), for a slice location matching the T_1_-mapping slice location. In subjects with AFD or PH, a minimum RVI of 4 mm was selected as an inclusion criterion for further analysis. This RVI thickness was selected for the T_1_-mapping data to mitigate potential partial volume contamination of myocardial tissue T_1_ values from neighbouring epicardial fat and/or blood pool pixels, based on a typical non-interpolated in plane resolution of approximately 1.9 mm. As healthy individuals were not expected to have increased RV wall thickness, their LV T_1_-mapping data was included if the septal or inferior LV wall thickness was at least 4 mm.

**Fig 2:**
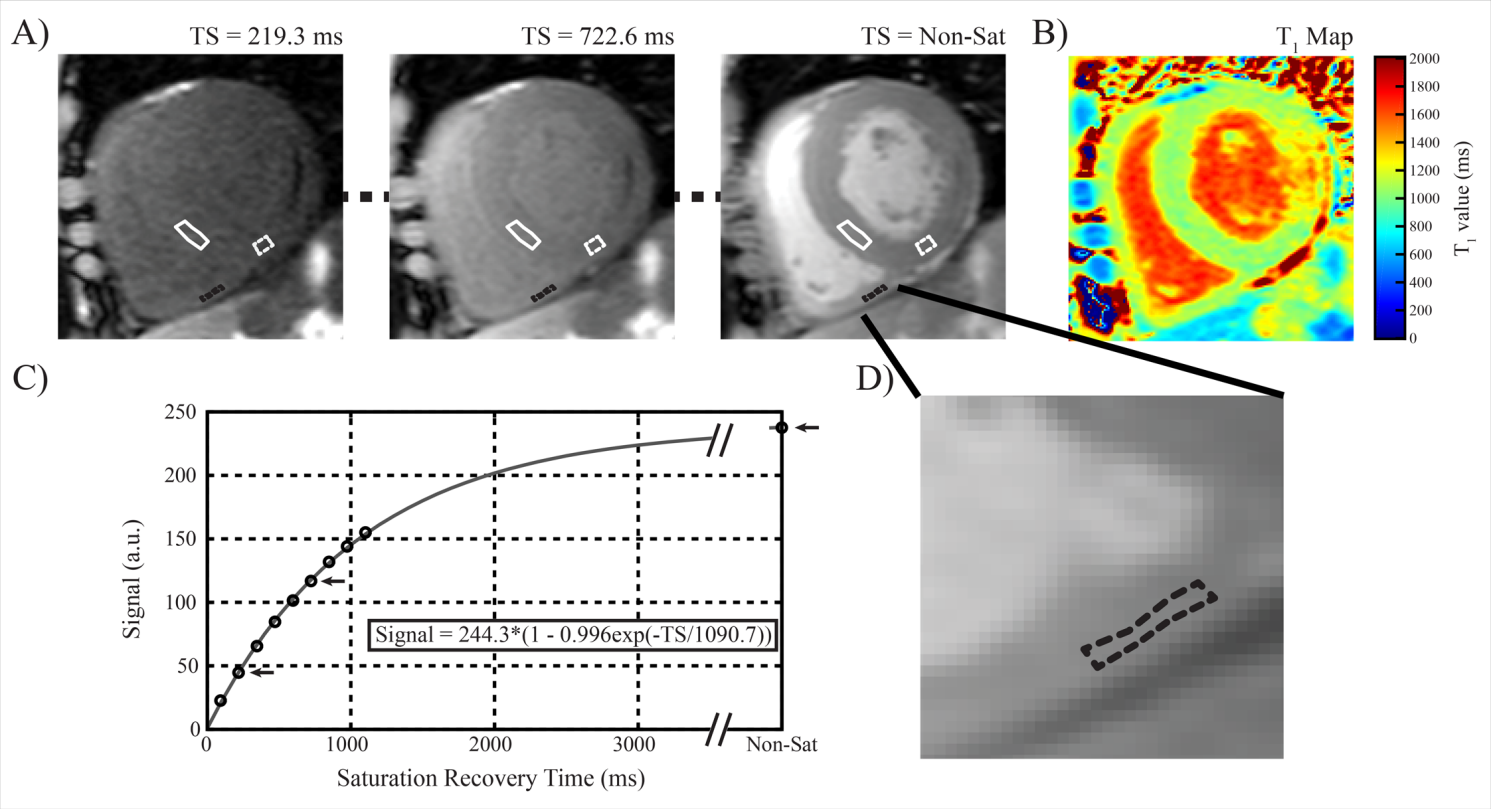
Example SASHA T_1_-mapping case. A) Sample SASHA T_1_-mapping images in a subject with Anderson-Fabry disease, showing septal (solid white), inferior right ventricle (dashed black), and inferior left ventricle (dashed white) regions of interest (ROI). B) Sample T_1_ map is shown. C) Mean signal intensity from the right ventricle ROI from this subject are plotted with the corresponding saturation recovery time (TS), along with a best-fit saturation recovery curve defined by the displayed equation. Black arrows indicate the saturation recovery images shown in A). D) A zoomed in portion showing the inferior RV wall, along with the corresponding ROI. Note the absence of visible pericardial fat in this location. The dark signal in the left ventricle lateral wall is a site of positive late gadolinium enhancement.

In all healthy controls, along with subjects with AFD or PH who met the criteria for minimum inferior RV wall thickness of 4 mm, the 10 images in the T_1_-mapping acquisition were registered to correct for in-plane motion occurring during the breath hold[24, 25]. Region of interest (ROI) tracing was then completed on the inferior RV wall, interventricular septum, and inferior LV wall with sample ROI placements shown in Fig 2. Care was taken to minimize potential contamination by blood or epicardial fat by avoiding the endocardial and epicardial borders, as well as avoiding the RV insertion point where positive LGE is commonly observed, particularly in PH[26]. Manual adjustment of ROI placement on the 10 images was performed if residual motion was noted following image registration. Signal intensities within ROIs were averaged prior to fitting a 3-parameter mono-exponential recovery curve, S(TS) = k(1- η exp(-TS/T_1_)), where k denotes a scaling constant, η represents the saturation efficiency, TS represents the saturation recovery time, and T_1_ represents the longitudinal relaxation time. For the assessment of reproducibility, analysis was performed on each subject twice, independently by two observers (JP and RBT). Subjects’ images were loaded randomly, with blinding between repeated observations and observers. T_1_ analysis was performed offline using custom software (MATLAB R2012a, The MathWorks, Natick, MA, USA).

### Statistical Analysis

Subject characteristics and CMR variables, including wall thickness and ventricular T_1_ values are presented as mean±standard deviation. Group differences, except with respect to gender, were compared using Kruskal-Wallis One-Way ANOVA, with multiple pair-wise comparisons performed using the Mann-Whitney U Test. Gender differences between groups were compared using Chi-squared analysis. Ventricular T_1_ values within subjects were compared using the Friedman test, with multiple pair-wise comparisons performed using the Wilcoxon signed rank test. Reliability was measured using Coefficient of Variation (CoV), defined as the standard deviation of the differences between repeated measurements divided by the measurement mean. Significance was set at p<0.05, with a Bonferroni correction where applicable. Statistical analysis was performed using STATA statistical software (Version 11.2, Stata Corporation, College Station, TX, USA).

## Results

Of the subjects included initially in the study, 6 subjects with AFD, 7 subjects with PH, and 0 healthy controls had inferior RVI thickness ≥ 4 mm and were therefore included for further T_1_-mapping analysis. One subject with PH had unanalyzable T_1_ datasets due to significant image artifact and was excluded and two HC subjects LVI T_1_ values were excluded due to LVI thickness < 4 mm. The characteristics of those included in T_1_ analysis are included in Table 1. Four of the six subjects with AFD were on enzyme replacement therapy, for a minimum of 4 years. Values derived from CMR scans are presented in Table 2. The average RVI thickness was not different between those with AFD and PH, but was thinner in HC than both AFD and PH (adjusted p<0.05, respectively). The average LVI thickness was larger in those with AFD compared to both PH and HC (adjusted p<0.05, respectively), while the IVS thickness was different between all groups, with the largest in AFD and the thinnest in HC (adjusted p<0.05 for all comparisons).

**Table 1:** Subject characteristics.

	AFD	PH	HC
	(n = 6)	(n = 6)	(n = 21)
Age (yrs)	46.7±8.1	49.7±17.6	40.5±15.9
Gender (M)	4	1	10
Weight (kg)	74.9±18.0	84.4±20.8	70.5±15.9
Height (m)	1.7±0.1	1.7±0.1	1.7±0.1
BSA (m^2^)	1.9±0.3	2.0±0.3	1.8±0.2
BMI (kg/m^2^)	25.1±5.5	29.8±6.0	24.8±5.4
HR (bpm)	65.8±12.8	78.0±14.1	64.7±7.2

AFD = Anderson-Fabry disease; PH = pulmonary hypertension; HC = healthy control; BSA = body surface area; BMI = body mass index; HR = heart rate; bpm = beats per minute

**Table 2:** CMR variables.

	AFD	PH	HC
	(n = 6)	(n = 6)	(n = 21)
IVS thickness (mm)	13±2*,†	10±1*	8±2
LVI thickness (mm)	10±3*,†	5±1	6±1
LV mass (g/m^2^)	97.7±32.9*,†	50.8±4.0	58.2±12.4
LV EF (%)	67.5±7.4	63.0±7.1	62.2±5.3
LVEDVi (mL/m^2^)	81.7±14.7†	57.0±8.5*	77.2±15.6
LVESVi (mL/m^2^)	26.2±6.1	21.2±5.8	29.3±8.0
IVS-T_1_ (ms)	1053±41*,†	1280±123	1180±60
LVI-T_1_ (ms)	1072±44*,†	1274±57*	1183±45
RVI thickness (mm)	5±1*	6±2*	2±0
RV EF (%)	69.3±8.3†	37.8±6.9*	58.1±7.3
RVEDVi (mL/m^2^)	71.0±18.2	123.3±47.9*	76.7±20.7
RVESVi (mL/m^2^)	22.5±9.3†	79.0±37.3*	32.6±11.9
RVI-T_1_ (ms)	1096±49†	1239±41	

AFD = Anderson-Fabry disease; PH = pulmonary hypertension; HC = healthy control; RVI = inferior right ventricle wall; IVS = interventricular septum; LVI = inferior left ventricular wall; EF = ejection fraction; LVEDVi = left ventricular end-diastolic volume indexed; LVESVi = left ventricular end-systolic volume indexed; RVI = inferior right ventricular wall; RVEDVi = right ventricular end-diastolic volume indexed; RVESVi = right ventricular end-systolic volume indexed

*p<0.05 compared to HC

†p<0.05 AFD vs. PH

**Fig 3:**
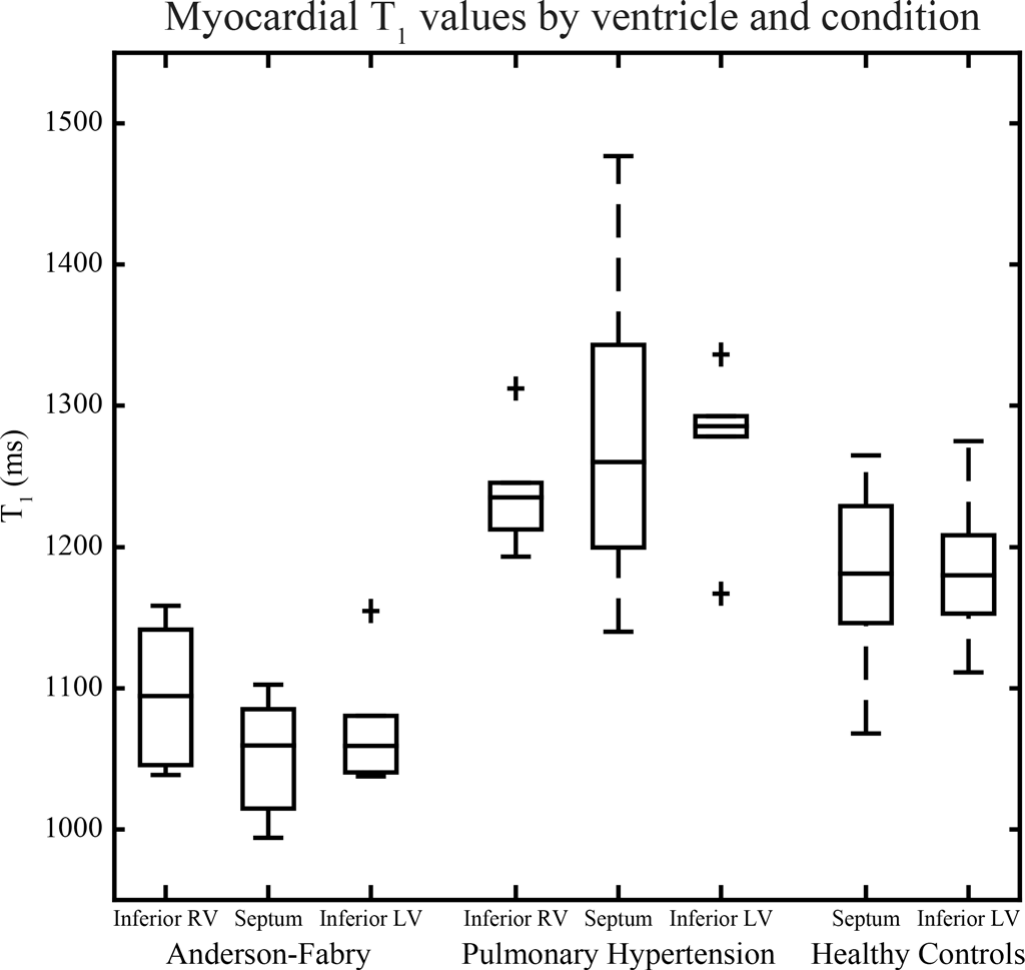
Myocardial T_1_ values by ventricle and condition. Data is presented as boxes representing the 25^th^, 50^th^, and 75^th^ percentiles, and fences representing 1.5× interquartile range.

As shown in Table 2, irrespective of ventricular location, myocardial T_1_ is shorter in subjects with AFD (RVI-T_1_ = 1096±49 ms, IVS-T_1_ = 1053±41 ms, LVI-T_1_ = 1072±44 ms) compared to those with PH (RVI-T_1_ = 1239±41 ms, IVS-T_1_ = 1280±123 ms, LVI-T_1_ = 1274±57 ms) and HC (IVS-T_1_ = 1180±60 ms, LVI-T_1_ = 1183±45 ms) (adjusted p<0.05 for all comparisons). Subjects with PH had longer LVI-T_1_ than HC (adjusted p = 0.02), but the IVS-T_1_ was not statistically different (adjusted p = 0.24), with a wide range of values (1140 to 1477 ms). T_1_ values for all groups are shown in Fig 3.

Comparing regions in subjects with AFD, T_1_ values were not statistically different between the two LV and RV locations (p = 0.31). Similarly, subjects with PH also showed non-significant differences between regional ventricular T_1_ values (p = 0.51).

Of the subjects with AFD who received contrast agents (n = 3), none were positive for LGE in the inferior RV, septal, or inferior LV regions. One subject showed positive enhancement at the inferior RV insertion point. One subject with PH showed inferior RV wall enhancement, 2 showed septal enhancement, and 1 showed enhancement at the inferior LV. All subjects with PH showed enhancement at the inferior RV insertion point.

Coefficient of Variability (CoV) for the repeated analyses by observer 1 was 1.4%, 0.9%, and 1.5% for the RVI, IVS, and LVI, respectively. For observer 2, the CoV was 1.2%, 0.6%, and 2.7% for the RVI, IVS, and LVI, respectively. For interobserver agreement, the CoV was 1.7%, 1.5%, and 1.8% for the RVI, IVS, and LVI, respectively.

## Discussion

The primary finding of the current study is the similar native T_1_ values in the right and left ventricle of patients with AFD and thickened right ventricles, both of which are reduced in comparison to LV T_1_ values in the healthy heart and as compared to patients with pulmonary hypertension. Reduced LV native T_1_ values were reported previously in patients with AFD[11–13], but this is the first report in the RV. Right ventricular involvement in subjects with AFD is common[17–20], typically manifesting as hypertrophy and/or myocardial dysfunction, and the findings of the current study suggest that the underlying changes in tissue characteristics are also similar in both ventricles. Autopsies studies have shown biventricular cellular hypertrophy, vacuolization and sphingolipid accumulation[21], thus the reduced RV T_1_ values in the current study likely reflect the same pathology and mechanisms as within the LV.

The exact cause and mechanism of the reduced native T_1_ in AFD values still requires further elucidation. Reduced LV T_1_ values have also been seen in individuals with iron overload[27], however this is unlikely the etiology in those with AFD. The effects of lipids on T_1_-mapping in general has not been systematically characterized, with one report of normal LV lipid content in AFD patients using ^1^H NMR spectroscopy[28]. Interestingly, a mixed RV response has been seen with enzyme replacement therapy[19, 20]. In a study by Wuest et al., 14 patients with AFD who received enzyme replacement therapy for approximately 1 year showed beneficial effects for both ventricles, including a decrease in RV mass and end-diastolic volume[20]. However, a study by Niemann et al. of 57 patients with AFD treated with enzyme replacement therapy for more than 3 years showed no decrease in RV wall thickness or end-diastolic dimension[19]. This suggests there may be additional factors involved in the development of RV hypertrophy in addition to deposition of sphingolipids or that therapy is less effective in the RV. The T_1_ differences noted in this study between AFD and PH suggests different origins of RV thickening between the conditions, indicating that RV thickening in AFD is not primarily related to increased RV afterload. Larger, and ideally longitudinal, studies including T_1_-mapping in the LV and RV would be useful in further elucidating the nature of RV involvement in subjects with AFD. Certainly, there is excitement in the community of the promise of T_1_-mapping, in either the LV or RV, to offer a potential biomarker to follow patients with AFD, particularly with respect to responses to enzyme replacement therapy.

In contrast, the increased native RV T_1_ values in PH (1239±41 ms versus 1096±49 ms in AFD) likely reflect fibrosis, or more specifically the increased water mobility associated with increased extracellular volume fraction, related to long standing pressure overload. In this study we also show that LV and RV native T_1_ values appear to be increased in those with PH and thickened RVs as compared to healthy control LV T_1_ values. There is limited existing literature to corroborate this finding, however a recent publication also showed increased native T_1_, along with extracellular volume fraction, in the RV of subjects with PH versus healthy controls[22]. Elevated native T_1_ values have been demonstrated at the RV insertion points in an animal model of chronic pulmonary hypertension[29], though septal values were not statistically different than controls. Our septal native T_1_ values were increased, though not statistically, likely reflecting pathological remodelling including a degree of interstitial and replacement fibrosis, evident by cases with positive late enhancement and reduced RV ejection fraction.

Quantitative T_1_-mapping, using native T_1_ values, has been used for non-invasive tissue characterization in a variety of disease conditions[14–16]; however, its use has generally been restricted to the left ventricle due in part to the limitations of spatial resolution. T_1_-mapping sequences typically have an in-plane spatial resolution of approximately 2 mm, thus making it difficult to apply in the right ventricle, where, in normal hearts the mean diastolic wall thickness of the RV is ≤ 4–5 mm[30, 31], compared to 6–10 mm for the interventricular septum[31]. The most relevant data comes from studies by Mehta et al., using the accelerated and navigator-gated look-locker imaging for cardiac T_1_ estimation (ANGIE) technique[22, 32]. Using this advanced technique, including optional fat-saturation, high-resolution RV T_1_-mapping was performed at end-systole, the authors found healthy control RV T_1_ and extracellular volume fraction values are similar to those found in the LV. Unfortunately, the high-resolution ANGIE sequence takes much longer than standard T_1_-mapping sequences (~3 minutes vs. 9–17 heart beats), may still be subject to partial volume contamination effects, and more practically is not yet widely available.

In the present study, the effects of partial volume contamination by either blood or epicardial fat was mitigated by selecting individuals with an increased RVI thickness, which is a cardinal feature of patients with Anderson-Fabry disease and pulmonary hypertension (5±1 mm and 7±2 mm, respectively, in the current study). Importantly, both patient groups’ RVI thickness were similar to the inferior LV thickness of healthy controls (6±1 mm), suggesting a similar small risk of partial volume contamination for parts of the LV. Subjects with PH were shown to have elevated T_1_ values in the LV and RV, while those with Anderson-Fabry disease have T_1_ values that are reduced in both ventricles. The primary technical concern with RV tissue characterization is systematic artifactual bias of these values from partial volume errors with epicardial fat or blood pool (which would be expected to decrease or increase T_1_ values, respectively). The consistently increased native T_1_ values in the PH group and reduced values in the AFD group, as compared to controls, and the excellent reproducibility of T_1_ values by two observers in the blinded analysis suggest that partial volume errors did not confound the major findings of the current study. Nonetheless, in the absence of robust techniques to remove the signal from blood and fat, the potential for signal contamination remains.

Our study is primarily limited by its small numbers and lack of normal RV T_1_ values for comparison. However, Mehta et al., showed similar normal LV and RV native T_1_ values using a high spatial resolution fat-supressed method[22, 32], suggesting our healthy control LV values may be used as a reference surrogate. Unfortunately, due to spatial and temporal resolution limitations of currently available T_1_-mapping methods, we limited our investigation to patients with thickened RV walls and therefore were unable to study the RV of healthy volunteers. This fact also limits the generalizability of our findings to other patients with Anderson-Fabry disease or pulmonary hypertension without a thickened RV. Also, while the inferior RV wall was consistently the most clearly discernible location with least amount of trabeculation and epicardial fat content (as observed on bSSFP cines), leading to our decision to perform analysis in that location, performing analysis on a small region at a single slice location may not accurately represent the entire ventricle. There is currently no data to suggest that there are intrinsic differences in native T_1_ values regionally within the RV.

Unfortunately, LGE imaging was not performed in all of the subjects with Anderson-Fabry disease so replacement fibrosis, and its potential to alter our reported T_1_ values, could not be ruled out. Previous reports typically describe elevated native T_1_ values in areas of positive LGE[11–13] where the basal inferolateral wall is most typically affected[33–35]. Thus, the reduced values seen in those with Anderson-Fabry disease in this study would not be typical if the ROI included a region with positive enhancement. However, we cannot rule out replacement fibrosis in other regions of the RV, where T_1_ analysis was not performed. Due to the lack of contrast administration, we are also not able to provide extracellular volume fraction estimates, which require native and post-contrast T_1_ measurement in the blood and tissue for calculation.

Further study, including clinical and function correlates, would add important diagnostic and prognostic significance to T_1_ measurements, however without significant improvements to T_1_-mapping techniques, larger scale RV imaging studies are not feasible.

## Conclusion

Though normal values for native T_1_ in the RV are still unknown, native T_1_ values appear similarly reduced in the left and right ventricles of individuals with AFD and right ventricular wall thickening, likely driven by the same pathological processes. In contrast, individuals with pulmonary hypertension and right ventricular wall thickening show increased native T_1_ values in both the LV and RV, suggestive of fibrosis. While T_1_-mapping is emerging as a useful marker in the diagnosis and monitoring of numerous cardiac conditions, its use in the right ventricle remains a challenge given this ventricles relative thinness and potential for blood pool or epicardial fat contamination.

## Supporting Information

S1 File Manuscript data. (XLSX)
